# U Can Touch This: How Tablets Can Be Used to Study Cognitive Development

**DOI:** 10.3389/fpsyg.2016.01021

**Published:** 2016-07-07

**Authors:** Kilian Semmelmann, Marisa Nordt, Katharina Sommer, Rebecka Röhnke, Luzie Mount, Helen Prüfer, Sophia Terwiel, Tobias W. Meissner, Kami Koldewyn, Sarah Weigelt

**Affiliations:** ^1^Department of Developmental Neuropsychology, Institute of Psychology, Ruhr-University BochumBochum, Germany; ^2^School of Psychology, Bangor UniversityBangor, UK

**Keywords:** tablet, touch screen, developmental psychology, methodology, children, memory, perception, recognition

## Abstract

New technological devices, particularly those with touch screens, have become virtually omnipresent over the last decade. Practically from birth, children are now surrounded by smart phones and tablets. Despite being our constant companions, little is known about whether these tools can be used not only for entertainment, but also to collect reliable scientific data. Tablets may prove particularly useful for collecting behavioral data from those children (1–10 years), who are, for the most part, too old for studies based on looking times and too young for classical psychophysical testing. Here, we analyzed data from six studies that utilized touch screen tablets to deliver experimental paradigms in developmental psychology. In studies 1 and 2, we employed a simple sorting and recall task with children from the ages of 2–8. Study 3 (ages 9 and 10) extended these tasks by increasing the difficulty of the stimuli and adding a staircase-based perception task. A visual search paradigm was used in study 4 (ages 2–5), while 1- to 3-year-olds were presented with an extinction learning task in study 5. In study 6, we used a simple visuo-spatial paradigm to obtain more details about the distribution of reaction times on touch screens over all ages. We collected data from adult participants in each study as well, for comparison purposes. We analyzed these data sets in regard to four metrics: self-reported tablet usage, completeness of data, accuracy of responses and response times. In sum, we found that children from the age of two onwards are very capable of interacting with tablets, are able to understand the respective tasks and are able to use tablets to register their answers accordingly. Results from all studies reiterated the advantages of data collection through tablets: ease of use, high portability, low-cost, and high levels of engagement for children. We illustrate the great potential of conducting psychological studies in young children using tablets, and also discuss both methodological challenges and their potential solutions.

## Introduction

Nowadays, new technologies accompany us nearly every second of our life. This is especially true of devices with touch screens, like smartphones or tablets, which have become our almost constant companions. This is not just the case for adults. Children too are not only fascinated by these devices, but are also able to easily access them due to the absence of any additional input requirements like mice or keyboards. For example, Cristia and Seidl (2015) report that about a third of children aged 5–11 months already have at least a monthly interaction with touch screens. This contact rises to almost 90% by the age of 3. Children this young can already tap (71%), flick (68%), drag (41%), and more. Indeed, Abdul Aziz et al. (2013) found that 2-year-olds can already tap and drag, while 3-year-olds also rotate and flick, and 4-year-olds can perform seven common touch screen gestures without difficulty. While these investigations focused on the general ability to interact with a touch screen, several areas of science have approached the use of touch screen tablets through more specific paradigms. In education, for example, Couse and Chen (2010) argue that interaction with tablets in the class room is viable: Children between the age of 3 and 6 are found to be curious about the new technology and “persisted without frustration” when learning to use them. Importantly, this active interest actually seems to carry over to increased learning. Neumann (2014), in a study investigating the effects of tablet use on literacy knowledge, found that at ages between 3 and 5, children showed improved letter sound and name writing skills when they had greater access to tablets. Having access to tablets was also found to be advantageous in a study by Hourcade et al. (2012) about the pro-social behavior in children with autism spectrum disorders (ASD). They provided children with ASD (age 5–14) with touch-screen-based applications and found that the mere use of this technology improved collaboration between children and provided a novel way to children with ASD to express their feelings. In a more general approach, Sobel et al. (2016) developed a tablet-based application that focused on promoting the inclusion of children with mixed abilities when playing with children without impairments. In short, they found that technology-forced interaction could improve cooperation between children pairs with and without disabilities. To help these advances, standardized testing (e.g., Luciana et al., 1999) is already employed by touch-screen-mediated technology since several years. Generally speaking, both parents (e.g., Neumann, 2014) and scientists (e.g., Christakis, 2014) seem to have a positive attitude toward touch screen technology and its effects on cognitive development and/or its use as a mediator of knowledge.

In developmental psychology, tablet-based experimentation has the potential to solve the challenge of the methodological gap between video-based preferential looking tasks and standard psychophysical experimentation. The former is often used with infants and toddlers (e.g., Delle et al., 2015), as they lack the necessary motor development to produce reliable, distinct and measurable physical responses to stimuli. But as these are purely passive tasks, young children from the age of 2 upwards are quickly bored when presented with the same paradigm over and over (e.g., multiple trials of the same task). On the other hand, children this young generally lack the necessary concentration and persistence to complete classical psychophysical paradigms, which have many trials and are often monotonous and repetitive. In many areas of developmental research, scientists have resorted to creative interactive experiments, for example using role plays (Warneken and Tomasello, 2008) or physical stimuli (Meltzoff, 1988). Unfortunately, such paradigms are often hard to quantify and difficult to conduct on a larger scale because of their labor-intensive nature during both data collection and analysis.

Furthermore, when investigating questions in the field of perceptual development through computerized measures one issue, that is prevalent in younger children, is response matching. By giving an answer through a mouse click or pressing a key to a stimulus on the screen, young participants often feel the need to physically look toward the input device and back onto the presentation device to match their response with the correct position on the monitor, what makes the process rife with errors. Additionally, these approaches require participants to be generally able to operate a computer and its input devices, which is of particular difficulty in children below the age of 5. Here, some studies test children (e.g., Suhrke et al., 2015) in such a way, that the children only indicate their answers (e.g., by saying it out loud), while the experimenter gives the physical response. Obviously, this procedure is prone to errors due to miscommunication between experimenter and participant, might introduce severe experimenter's bias and lacks the possibility to record reaction times. Furthermore, work stations with equipment (monitor, mouse, keyboard, loudspeakers) are of a very stationary nature.

Touch screen tablets could help with these issues. On the one hand, the computerized, digital data conduction would allow for a more neutral, bias-free recording and easier analyses compared to role plays or physical constructs. But more importantly, due to the employment of tablets as paradigm mediators, large-scale parallel data acquisition could be realized by having young participants directly interact with the experiments, compared to the need for lengthy one-on-one sittings with current methods. Additionally, in areas, in which education is combined with a high number of children, such as museums, kindergartens, and schools, data conduction could be swift, comfortable, and rewarding for both parties.

To investigate their potential, Frank et al. (2016) very recently conducted a first study to test the general viability of tablets in developmental cognitive research in children (age 1–4). They compared three methods of measuring response during a word-recognition paradigm: presentation on a web-technology-based tablet, a storybook method and an eye-tracking paradigm. Their results showed the tablet to be on par or even favorable to the other methods in reliability, performance and sensitivity of reaction times, thus arguing in favor of adopting tablet-based paradigms as a viable new research method.

Taken together, initial evidence suggests utilizing tablets might help to fill the aforementioned methodological gap in developmental research: their high accessibility, ease of use, relatively low cost and accurate, digital measurement abilities provide everything needed to successfully conduct cognitive experiments with young children. Additionally, Frank et al. pointed out that tablets both increase the accessibility of special populations and remove some sources of experimenter bias through computerized stimulus presentation. Thus, utilizing tablets holds promise for allowing researchers to not only collect larger data sets more quickly but also to refine currently established methods. Here, we test the viability of using touch screen tablets in the study of cognitive development. We aim to identify potential limits regarding necessary motor skills and/or the maximal complexity and duration a psychological research paradigm may have for children in particular age groups when the experiments are mediated through a tablet.

In this study we analyzed six data sets, collected through independent tablet-based cognitive experiments conducted with adults as well as children between the ages of 1 and 10 years (see Table 1 for an overview). Data sets were acquired through a variety of perception, learning, and memory tasks commonly used in adult cognitive psychology research, including sorting tasks, 2-alternative forced choice (2AFC) memory tasks, 2AFC-perception tasks, a visual search task, an extinction learning paradigm and a task for assessing spatio-temporal accuracy. Each study consisted of both a sample of adults and a sample of children. While the age of the children was dependent on the task, adults were aged between 18 and 37 in all studies. Briefly, the first two studies consisted of a two-option sorting task followed by a memory task. In both studies, stimuli had to be categorized in the first step before being recognized in a subsequent 2AFC recall task (shorthand: Sort Recall). In general, tasks in the field of perceptual development are designed as 2AFC tests as they allow for a clear differentiation between the intended responses, even in a young age. The studies differed in their level of difficulty; the first was easier (designed for children aged 2–5) while the second used more difficult stimuli (designed for children age 4–8). The third study extended the same sort of paradigm by adding bodies to the car and face stimuli included in studies 1 and 2. The study also employed an additional task, a set of staircase-based 2AFC perception tasks using the same types of stimuli as were presented in the memory task (shorthand: Sort Recall Perception). These two modifications increased the difficulty of the paradigm quite a bit (designed for children aged 9–10). The fourth study (shorthand: Visual Search) was a viewpoint-dependent visual search task with faces and cars as targets among object distractors arranged in a 3 × 3 grid, designed for children aged 2–5 years. The fifth study (shorthand: Extinction Learning) investigated an extinction learning paradigm in 1- to 3-year-old children. Here, some of the upwards flying balloons were only “poppable” in the learning and renewal phases (indicated through colors), while the rest were poppable throughout the whole experiment. The sixth study consisted of a spatio-temporal accuracy measurement (shorthand: Visuo Spatial RT) to obtain a baseline measurement of spatio-temporal abilities that could be used to “correct” response times across all experiments (i.e., are 3-year-olds slower than 5-year-olds when spatio-temporal skills are taken into account?). Here, the stimulus differed across trials in position and size and participants had to react as quickly as possible by touching it on the screen. This data was collected from the same participants as those in studies 3, 4, and 5; thus, this data set included children aged between 1 and 10 as well as data from the adult participants from studies 1, 3, 4, and 5.

**Table 1:** **Overview over all studies**.

	**study name / tasks**	**Response**	**Stimuli**	**Age Range**	**Duration**
1	Sort Recall easy	drag and drop	faces, cars	2–5 years, adults	15 min
2	Sort Recall difficult	drag and drop	faces, cars	4–8 years, adults	20 min
3	Sort Recall Perception	drag and drop	faces, cars, bodies	9–10 years, adults	35 min
4	Visual Search	tap	faces and cars among objects	2–5 years, adults	15 min
5	Extinction Learning	tap	moving balloons	1–3 years, adults	5 min
6	Visuo Spatial RT	tap	static green frog	1–5 years, 9–10 years, adults	2 min

*The table lists the studies we analyzed in this work along with basic details about each. Details for each study can be found in the methods section*.

To assess how well children can interact with tablet-based paradigms from cognitive psychology, we analyzed each study using four metrics: Usage, completeness, accuracy, and response time. This step-wise approach allowed us to analyze more finely-grained information with each subsequent metric. First, we used a simple questionnaire item to assess the prevalence of tablet use in participants, thereby allowing us to measure how tablet familiarity might change across different age-groups. Second, we checked how much of each experiment was completed by our participants. By gathering this metric, we assessed at which age children had the necessary motor skills to complete the task, as well as by which age children had the necessary motivation and endurance to complete all the trials included. If children of a particular age tended to quit an experiment early, we can infer that the experiment needs to be shorter or more entertaining to adequately engage that age group. Additionally, if there were very low rates of completion at specific ages, the complexity of the task—either on a cognitive or motor level—might be too high for use with children at that age. Cognitive requirements were further investigated through the third metric, accuracy. In this next step, data sets were checked for a high amount of error, independent of task-specific questions. Obviously, an interaction of accuracy and age is expected, as the studies in question all target age ranges during which the respective cognitive traits are thought to be developing. Despite this, performance in any age group should not be at either chance or ceiling level. Chance or ceiling performance in any group demonstrates a difficulty that is too high or low for a certain age. At chance levels, participants might have resorted to guessing, while no task-specific effects can be found when ceiling results are present. Lastly, we analyzed the data with regard to reaction times to identify potential age-dependent increases in speed. Thereby we were able to complement previous analyses and infer potential limitations when designing further psychophysical experiments on touch screen tablets.

Additionally, by comparing the results of each metric subsequently to adult data, we will be able to identify potential age thresholds, at which children data compares to adults. Identifying these developmental differences allows employing a guideline at which age experimental paradigms are viable, either by providing a difference in accuracy or by having comparable reaction times. Taken together, this study aspires to establish a first basis of the kinds of paradigms and experimental parameters which can be successfully conducted through tablet experimentation in developmental psychology.

## Methods

### Participants

Participants were mainly recruited through visits to day care centers, kindergartens and schools in the Rhein-Ruhr area in Germany and at the Ruhr-Universität Bochum with regard to adult participants. Each participant and/or his legal guardian signed a consent form before participating. Adult participants participated out of good will or were rewarded with course credit, while children were allowed to choose from a variety of small toys after participation, regardless of completion of the experiments. Ethical approval was obtained from the local ethics board for each study.

In study 1 (Sort Recall easy), one participant was removed from the analysis due to technical issues, yielding 93 data sets. Of those, 79 participants were children in the age range of 2–5 (*M* = 3.43, *SD* = 1.15). Across these data sets, four single answers were corrected where the participant very clearly indicated that (s)he intended to choose a different stimulus after his/her decision, thereby changing three answers from “error” to “correct” and one the other way around. To prevent this issue, the arrangement of the task was changed in later studies (see 0 for details). The 14 adult participants were on average 21.21 (*SD* = 2.42, range = 19–28) years old. In study 2 (Sort Recall difficult) we had to exclude two of 77 participants due to technical issues, yielding 75 usable data sets. The mean age of the remaining 65 young participants was 5.88 (*SD* = 1.39, range = 2–8 years), while those of the 10 adults was 21.6 (range = 19–24 years, *SD* = 1.71). Study 3 (Sort Recall Perception) consisted of 36 participants, where 20 where in the range of 9–10 years (*M* = 9.65, *SD* = 0.49) and 16 were adults in the range of 19–30 years (*M* = 22.81, *SD* = 3.45). Of 107 data sets in study 4 (Visual Search), two had to be excluded due to visual impairment of the participants and an additional two due to missing questionnaire data. The remaining 103 participants consisted of 86 2- to 5-year-old children (*M* = 3.8, *SD* = 0.97) and 17 adults in the range from 20 to 37 years (*M* = 24.12, *SD* = 4.39). In study 5 (ExctinctionLearning), two of 64 participants were excluded because of technical issues, thus we were able to analyze 62 data sets. Of those, 46 children from 1 to 3 years participated (*M* = 1.76, *SD* = 0.67) along with 16 adults in the range of 18–30 years (*M* = 23, *SD* = 3.33). Participants of study 6 (Visuo Spatial RT) consisted of adult participants of studies 1, as well as all participants from studies 3, 4, and 5, a total of 217 data sets. Of those, seven participants had to be excluded due to technical issues, while one was excluded as there was no age given on the participation form. The remaining 209 participants were divided in 150 children from 1 to 10 years (*M* = 4.05, *SD* = 2.52) and 59 adults in the age range of 18–37 years (*M* = 22.32, *SD* = 3.47). Some adult participants took part in multiple studies, but were never shown the same stimulus material more than once. For details on the age distribution for each study, please refer to Table 2.

**Table 2:** **Summary of participants**.

	**1**	**2**	**3**	**4**	**5**	**6**	**7**	**8**	**9**	**10**	**Adult**	**Σ**
Study 1 (Sort Recall easy)		24	15	22	18						14	93
Study 2 (Sort Recall difficult)				14	14	13	14	10			10	75
Study 3 (Sort Recall Perception)									7	13	16	36
Study 4 (Visual Search)		9	23	30	24						17	103
Study 5 (Extinction Learning)	17	23	6								16	62
Study 6 (Visuo Spatial RT)	15	27	32	30	26				7	13	59	209

*A list of the number of participants over all studies, presented per age*.

### Hardware and software

Studies 1, 2, and 3 used an Acer Iconia W510 tablet with a 10.1 inch screen, while studies 4 and 5 used an ASUS Transformer Book T300FA with a 12 inch screen (resolutions: 1366 × 768 px). Study 6 was conducted using both. With regard to the operating system, studies 1 (children data) and 2 (all data) ran on Windows 8, while all other studies ran on Windows 10. Due to the ease of implementation, we used web technology to show our stimuli and record the data. To remove the reliance on an internet connection and minimize data security concerns, we installed a local webserver. Each tablet had XAMPP 3.2 installed and ran PHP 5 on Apache 2.4. The front-end was mediated through Google Chrome and JavaScript aided by jQuery 2.1, jQuery mobile 1.4, and jQuery UI 1.10. The experiments were programmed in HTML5/JavaScript and presented full-screen. To increase the sensitivity of touch screens for very young children, all tablets were adjusted to have a higher sampling rate and lower sampling latency of touch events through a registry edit (decrease of parameters Latency and SampleTime from 8 to 4). Additionally, to minimize accidental resizing or navigation, we disabled some of Chrome's gesture features (“Overscroll history navigation” and “Enable Pinch”).

### Stimuli and design

#### Study 1: sort recall easy

**Figure 1:**
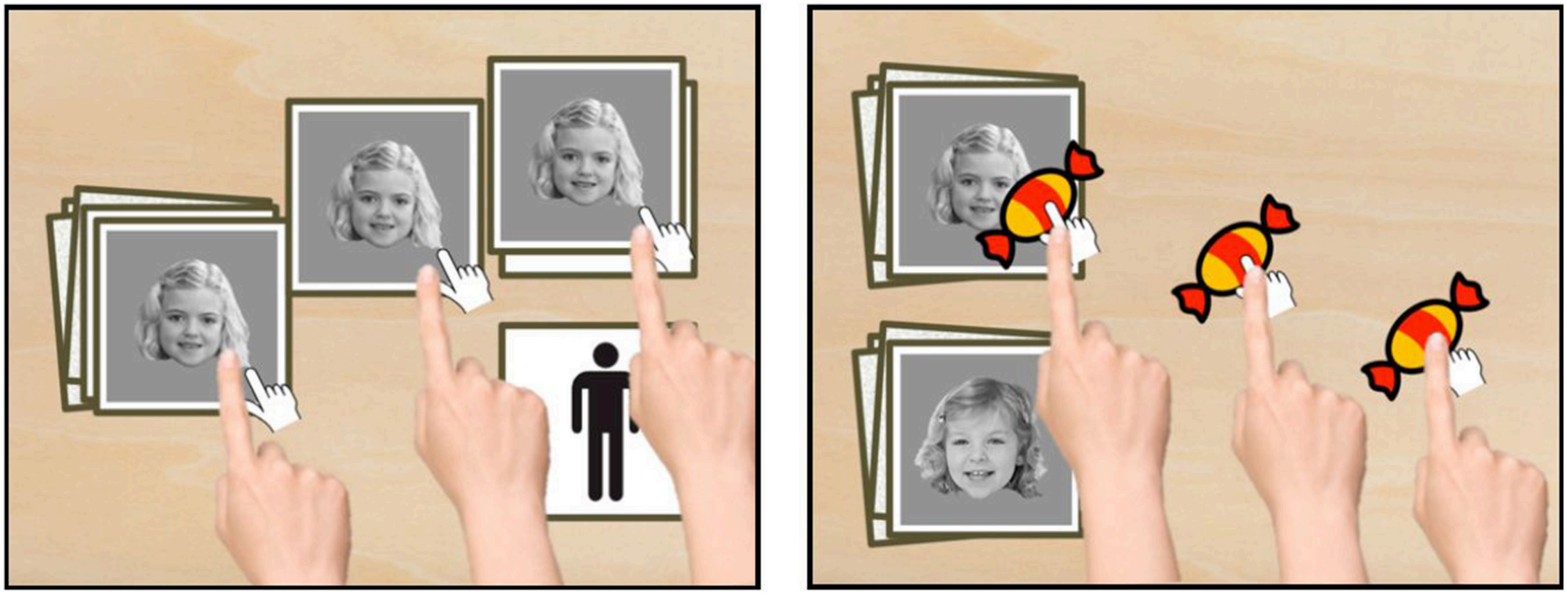
**Design of study 1 (Sort Recall easy)**. On the left, an example of a sorting trial (phase 1) can be seen. The image had to be dragged to the right side onto one of two available categories. On the right, an example of the recall task can be seen. Participants were instructed to drag the candy to the stimulus they recognized (“Which one have you seen before?”).

The first study employed a two-option sorting task followed by a 2AFC memory task to examine the development of facial recognition in children (e.g., Weigelt et al., 2014). In the first phase, participants tapped a stack of cards on the left side of the screen to reveal a stimulus. Following a 3000 ms delay, a small finger icon appeared to indicate the ability to categorize the card. Participants categorized the image through dragging and dropping the picture on the appropriate stack on the right side of the screen (see Figure 1, left). After all stimuli had been sorted, the memory task started. During the memory phase, participants revealed two stimuli on the left side of the screen (see Figure 1, right). After a 3000 ms delay, an image of a candy appeared that was also draggable. Participants were instructed to drag the candy to the image they had seen before. Each sorted and remembered image was followed by a short applause sound, regardless of the correctness of the decision. In total, three blocks were performed. The first block was a training block with four trials using dog and cat faces as stimuli, followed by a block of six faces of children (male/female), then a block of six cars (open/closed top). Within blocks, images were presented in a randomly selected order. The total duration of the experiment was ~15 min. Each image was 300 × 300 pixels and grayscaled. To better differentiate between the cognitive tasks of categorization and memory, analyses will be performed on each task separately. All images were taken from the Internet and modified to fit the experimental design.

#### Study 2: sort recall difficult

**Figure 2:**
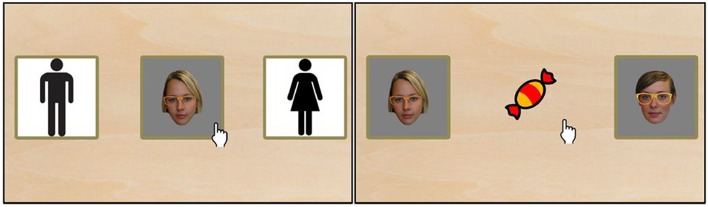
**Example trial from study 2 (Sort Recall difficult)**. On the left, the sorting stage is shown. Each face image had to be dragged to the corresponding category on either the left or right side of the screen to clearly differentiate between the intended motor action. Subsequently, as can be seen on the right, the candy had to be dragged to the already seen face.

The second study was an extension of Sort Recall easy, but investigated the influence of paraphernalia on facial recognition (e.g., Bulf et al., 2013). We changed the arrangement of the interact-able objects by moving one stack of cards to the left and one to the right side, while presenting the pictures to be sorted in the sorting task and the candy in the recall task in the middle of the screen (see Figure 2). This change was intended to reduce the possibility of accidentally misplacements, a scenario that is much more likely when objects are dragged in the same direction for both categories (as noted in the participants section). This new set-up also more equally distributes stimuli over the whole screen and allows for a clearer differentiation of the intended motor act as participants must decide to move toward either the right or left side of the screen. The stimulus set of study 2 contained full-color adult faces combined with added paraphernalia (hats and glasses). While the sorting task was equivalent to study 1, the memory task therefore allowed differentiating between five possible changes for the stimulus between sorting and recall: No paraphernalia, constant paraphernalia, removal of paraphernalia, added paraphernalia, and change of paraphernalia (for an exemplary trial, see Figure 2). The training block at the start of the task consisted of one trial that covered each of these five possibilities. The training block preceded two experimental blocks with 10 trials each. In total, the experiment took about 20 min. The modification of stimuli between sorting and recall phase increased the overall difficulty of study 2 compared to study 1, thus we increased the age range of child participants to 4–8 years. Each of the 40 faces (20 targets, 20 distractors, gender equiprobable) was taken from the Glasgow Unfamiliar Face Database (Burton et al., 2010), while hats and glasses were taken from various places of the Internet and adjusted to fit our needs.

#### Study 3: sort recall perception

**Figure 3:**
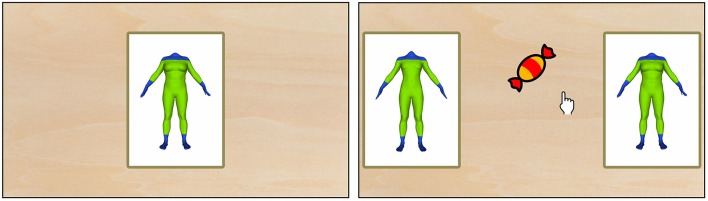
**Example perception task trial from study 3 (Sort Recall Perception)**. First, a stimulus was shown (left). Then, the participant was asked to drag the candy to the stimulus s(he) had seen immediately before. Each correct answer increased the similarity between target and distractor by 5%, while wrong answers decreased the similarity by 15%.

Extending the two previous studies, study 3 (Sort Recall Perception) covered two social and one Non-social stimuli types (faces of children, cars, bodies of adults; see Weigelt et al., 2014) and added a staircase-based 2AFC perception task. In the memory tasks, the same design and procedure as in study 2 was used but with different stimuli, shortening the delay to 1000 ms and removing the applause after each trial. The sizes of images were adjusted to better fit their natural proportions, i.e., cars being horizontally rectangular, bodies vertically rectangular and face images kept square. Bodies were clothed in skin-tight “super-hero” outfits, presented from the neck down and colored in bright green and blue to be more appealing to children (Figure 3). The perception task started with a centrally presented stimulus, followed by a 1500 ms delay and the subsequent presentation of two stimuli. The participant had to drag the candy to the item s(he) had seen immediately before. Each correct answer moved the distractor morph toward the target image, which was kept at 95% of the original stimulus and 5% of the distractor stimulus. Two staircases worked in a 1-up 3-down way in parallel by in-/decreasing the morphing between the two stimuli by 5% per step, thus increasing the similarity by 5% per correct and decreasing the similarity by 15% for each wrong answer. Each staircase ran until eight reversals were detected, where one reversal was defined as a wrong answer. Until the first reversal, errors in the first 25% did not result in a reversal. A minimum of 5% difference between target and distractor stimulus was enforced and trying to surpass that threshold through a correct answer was counted as a Non-error reversal, while keeping the stimulus values the same. Taken together, the three tasks took about 35 min to complete. Due to the large difference in task requirements, we split this study into its parts (sorting, memory, perception) and analyzed them accordingly. Due to much higher difficulty of the stimuli, especially in the perception task, only children from the age of 9–10 years and adults were tested. Faces were taken from the Dartmouth Database of Children's Faces (Dalrymple et al., 2013), body stimuli are 3 d mesh models created from full-body scans of adults (purchased from www.bodylabs.com) and cars were taken from various websites from the Internet.

#### Study 4: visual search

**Figure 4:**
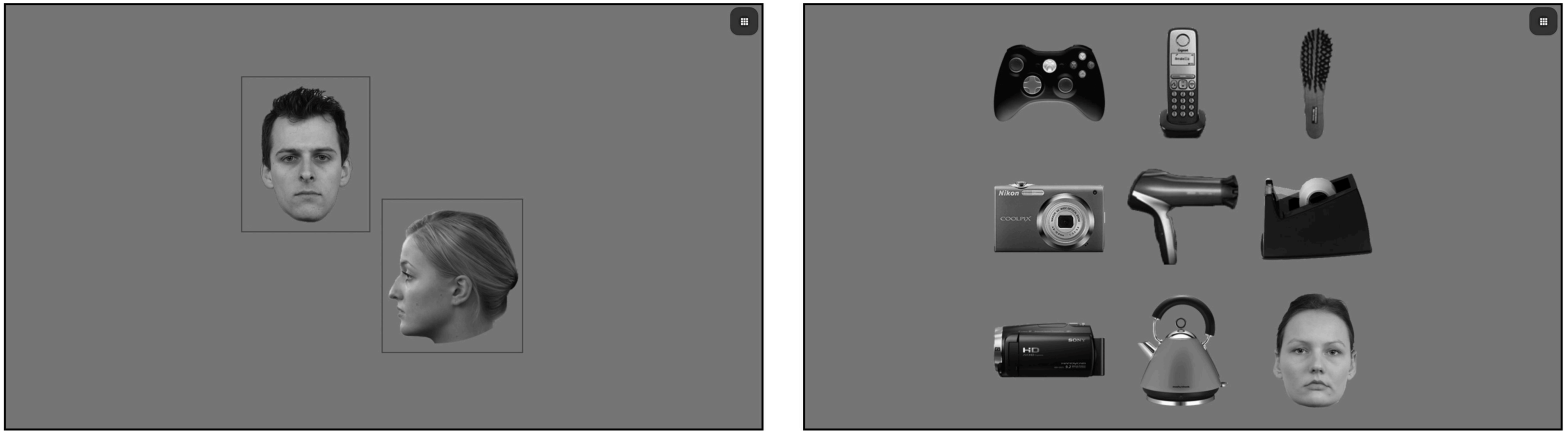
**Design of study 4 (Visual Search)**. At the start of each block, the target stimulus type is shown in 2 viewpoints, as can be seen on the left. Each trial consisted of an array of images containing one image from the same category (in this case faces) along with eight distractors in a grid. Participants were instructed to find and tap the target as quickly as possible.

Study 4 employed a visual search task with children from the age of 2–5 years (similar to Di Giorgio et al., 2012). Each block consisted of 10 trials and started with an image representative of the target type for that block, which were either faces or cars (see Figure 4, left) and ended with an applause sound. Each trial within the block started with a placeholder image that had to be tapped to reveal the test array. The target stimulus was presented at a random location in a 3 × 3 grid with eight distractors (see Figure 4, right). Upon tap on any of the images, the screen went blank for an ITI of 1000 ms before the next trial started. Two training blocks (three face trials and two car trials) preceded eight blocks of experimental trials. Each presented image was 250 × 250 px and randomly selected out of 720 possible items. In total, 40 faces (20 male, 20 female), 40 images of cars and 640 distractor images of various items roughly similar in size to the faces were used. Half of the target images were exhibited from the front while the other half were viewed from the side. Face images with a neutral facial expression were taken from the Radboud Faces Database (Langner et al., 2010), from the Karolinska Directed Emotional Faces database (Lundqvist et al., 1998) and from the Aging Mind database (Minear and Park, 2004). Photographs of cars and distractors were taken from the Internet and modified to fit our purpose. The whole task took about 15 min to complete, while after each block the participant was asked whether (s)he wanted to “continue playing the game.”

#### Study 5: extinction learning

**Figure 5:**
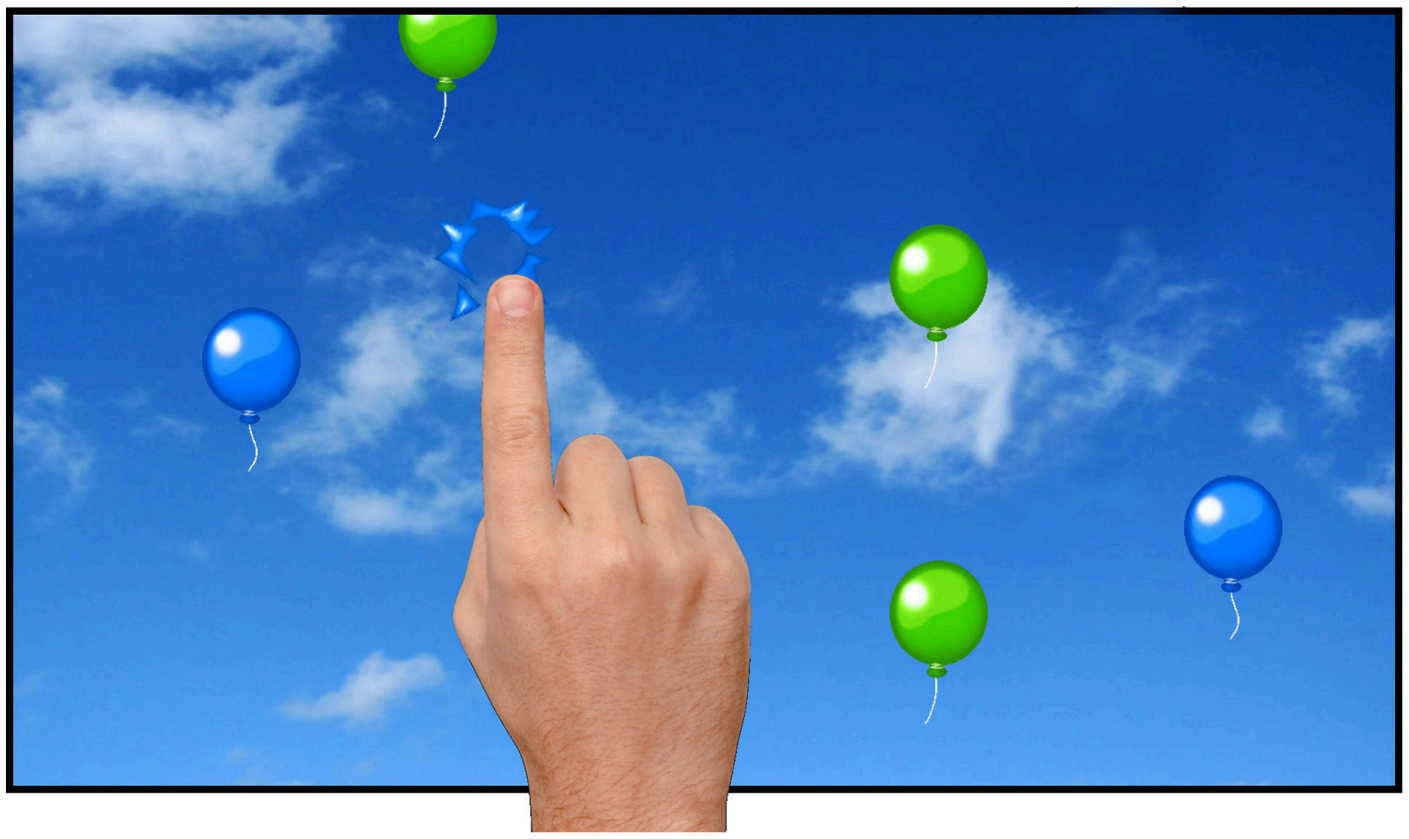
**Example trial from study 5 (Extinction Learning)**. Multiple balloons appeared on the screen and moved from the bottom of the screen to the top. In the learning and relearning conditions, all colors popped when being tapped, while in the extinction phase only a single color was poppable.

In study 5 an extinction learning paradigm consisting of three phases was performed (see Happaney and Zelazo, 2004) with children between the age of 1 and 3 years as well as with adults. In the first phase (learning), balloons of two colors ascended from the bottom of the screen to the top (Figure 5). Upon any of the balloons was tapped, the balloon popped and an accompanying sound was played. During the learning phase, all balloons (in two colors) were poppable. In the second phase (extinction), however, balloons were shown against a different background color (gray/blue), and only balloons of one color were poppable. In the third phase (renewal), the same settings as in the learning phase were used. For adults, the balloons were 200 × 330 px, two random colors were picked from the set of green, red, blue, and yellow, it took about 7800 ms for a balloon to reach the top, and there were six balloons on screen at any given moment. For children, parameters were adjusted to fit their ability after estimating their ability based both on prior participant data and the results of their own first block data. Thus, between two and six balloons were presented at the same time with an on-screen time between 7000 and 13,000 ms. Each block took 90 s, summing up to an experimental time of 5 min.

#### Study 6: visuo spatial RT

**Figure 6:**
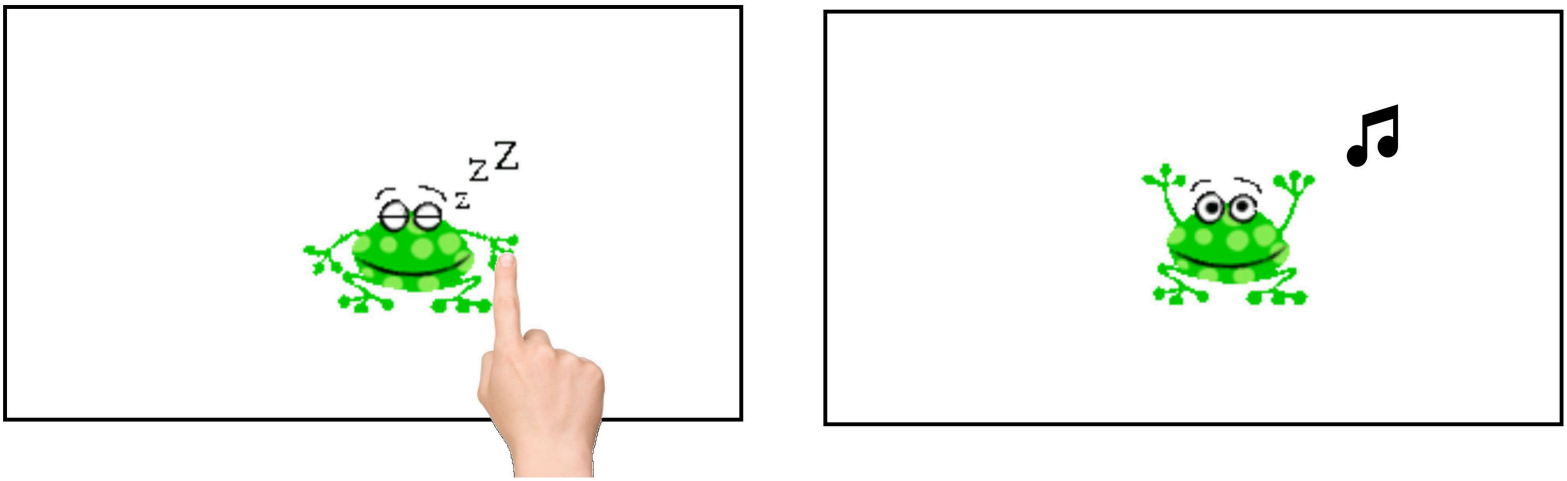
**Example trial from study 6 (Visuo Spatial RT)**. In each trial a sleeping frog appeared on the screen. When the frog was tapped, a sound was played and the frog jumped twice, before disappearing. Size and position of the frog varied across trials to get a more isolated measurement of tapping speed and accuracy without increasing cognitive load.

The final study we analyzed in this work was a simple visuo-spatial reaction time measurement. In this study, we intended to get a more general picture of the ability to use taps on a tablet without cognitive interference. Thus, a simple reaction time task was used where only the position and size of the stimulus varied. In each trial, a green sleeping frog appeared on the screen (see Figure 6, left). When tapped, the frog jumped twice before disappearing and a short sound was played. The full task consisted of four blocks of five trials each with 1000 ms ITI between trials. After three training trials, the frog first appeared centrally (condition 1), then appeared at random positions and decreased in size from 200 × 160 px (condition 2) to 100 × 80 px (condition 3) to 50 × 40 px (condition 4). If no tap was detected within 10 s, the trial was determined as “not tapped” and ended. The task took about 2 min to complete.

### Analysis

The metric “tablet usage” was simply determined through a questionnaire item on the consent form participants and/or their legal guardians signed before taking part in our studies. The question “How familiar are you with devices that have a touch screen (e.g., a mobile phone or tablet)” could be answered either with “no experience,” “little experience,” or “much experience.” To determine the “completeness” of studies, we used a mixture of observational data collected during testing and Post-test checking of each data set for missing data. First, each participant's data was checked for missing trials and a percentage of completed trials was calculated. Additionally, in each study, the experimenter noted when a child did not want to finish the study (e.g., boredom, fear of the stimuli or similar reasons). Those two factors combined yielded the relative completion rate of each participant. A special case was study 4 (Visual Search), where we expected children to only complete four of the eight experimental blocks due to the repetitiveness of the paradigm. Thus, four blocks was considered to constitute 100% completion; additional data was seen as optional icing on the cake. Because of this, as well as the fact that sometimes children wanted to repeat tasks (especially study 6), data might reflect completion greater than 100%. In such cases, we trimmed “completeness” down to 100%. In addition, for assessing completion rates, all data, including training trials, were used. How the metric “accuracy” was calculated depended on the study. In studies 1 (Sort Recall easy), 2 (Sort Recall difficult), 3 (Sort Recall Perception), and 4 (Visual Search), we were able to use the inverted error rate of each task as an accuracy measure for each participant. In study 5 (Extinction Learning), accuracy was calculated as the sum of hits and correct rejections compared to misses and (repeated) false alarms. As study 6 (Visuo Temporal RT) did not have “correct” and “incorrect” answers, we defined those trials where participants did not answer within the 10 s of presentation time as erroneous (missed trials). Training data was excluded when calculating accuracy rates, as experimenters often used training trials to explain the task to the children. Response times in studies 1, 2, and 3 were determined as the time between appearance of the stimulus and either dropping the stimulus on a categorization stack or dropping the candy on either of the images. In studies 4, 5, and 6, response time was determined between the appearance of the stimulus and either the tap on any of the nine images, on a balloon, or on the frog, respectively. To calculate response times, training trials and error trials were excluded; in the case of study 5, only hits were used. Subsequently, we compared the metrics completeness, accuracy, and reaction time to adult data to identify a potential convergence of children on adult data. This procedure allows inferring at what point it would be safe to assume an equal senso-motoric point of action when conducting tablet experiments. Furthermore, to avoid any observer-expectancy effects, the metrics and their calculation were not known to the experimenters who acquired the data, but only revealed after completion of data collection.

## Results

### Tablet usage

**Figure 7:**
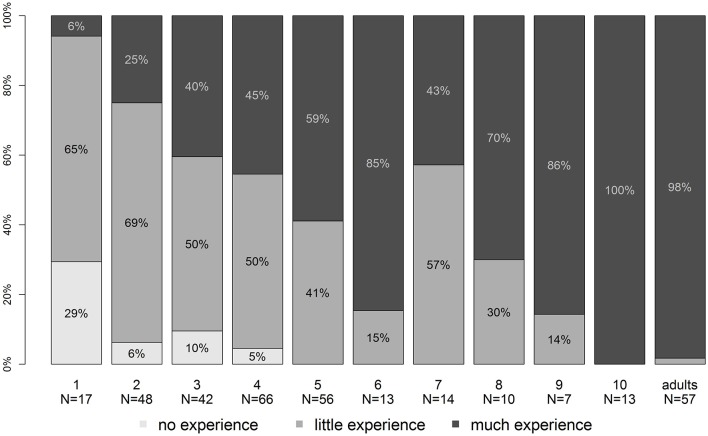
**Usage of tablets by participants**. The graph depicts self-reported experience with tablet-like devices by the participants or their legal guardians, plotted against age.

Figure 7 depicts the merged questionnaire data of all experiments. While 29% still did not have contact with tablets at the age of 2, that number steadily decreases until participants are 5 years old, where everyone had at least a little experience with touch screen devices. The regular use of such devices then increases, reaching a majority around the age of 8. In short, the older the children, the more prominent is tablet use up to an age of 10, where tablet usage reaches adult levels.

### Data completeness

**Figure 8:**
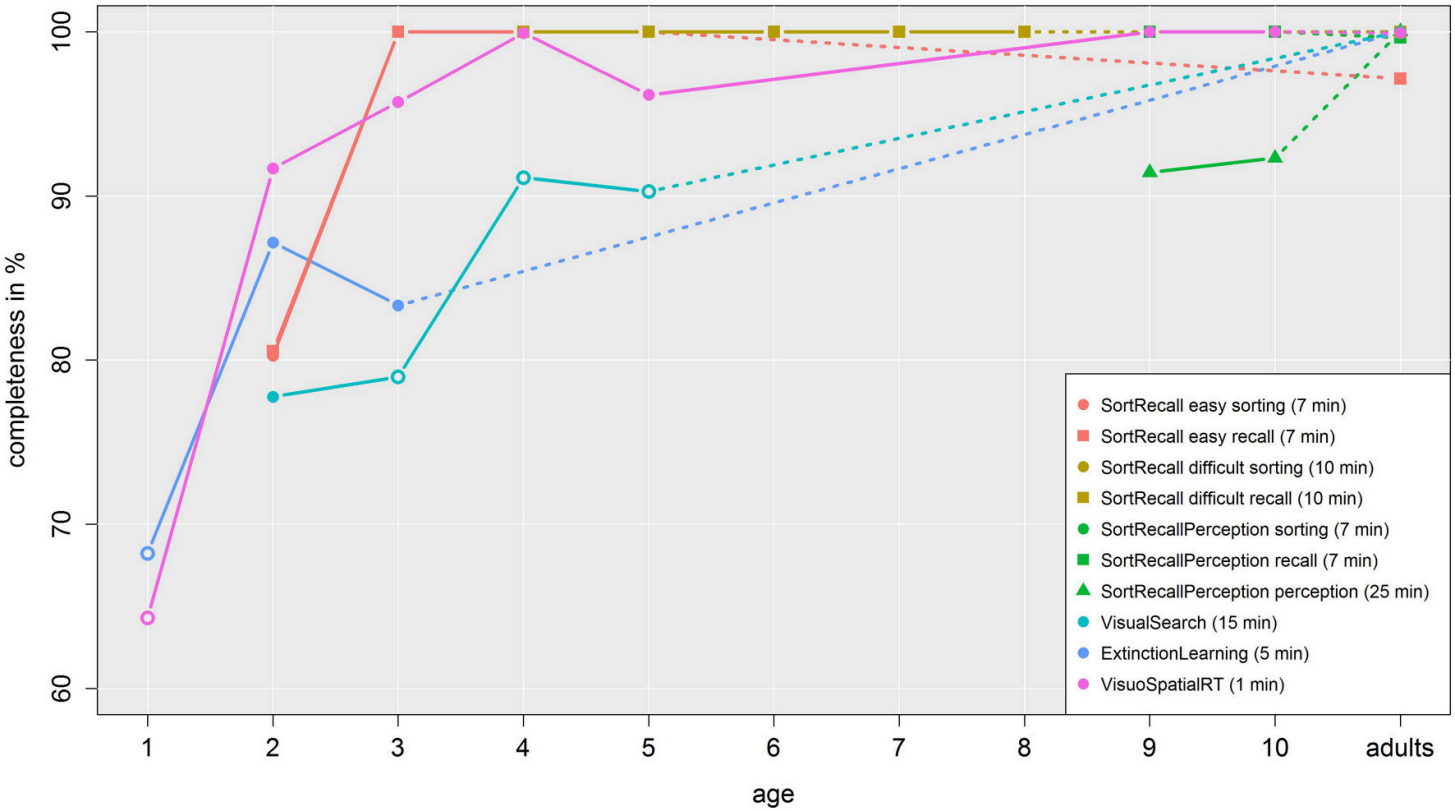
**Completeness of data**. This figure shows to what extent we were able to collect data at each age. Each line represents one task in one study, while the legend includes the duration of each task. The dashed line connects children with adult data. Empty symbols denote a significant difference between children and adult data.

In general, our studies were designed to allow our (young) participants to complete them. To investigate if we reached this goal, we plotted the percentage of complete data sets for each study in Figure 8. Each colored line represents one study; those with multiple tasks (Sort Recall easy, Sort Recall difficult, and Sort Recall Perception) are represented with one line of identical color for each sub-task but with a varying symbol. Child data is linked to adult data with a dashed line. On average, we were able to obtain around 64% of the data we intended to acquire from 1-year-olds, about 84% from 2-year-olds and 90% for 3-year-olds. By the age of 4, almost all participants finished all of the respective trials, regardless of study length. The only notable exception was the perception task in Sort Recall Perception. Here, most children that did not complete the entire set of tasks simply ran out of time (due to data acquisition being tied to the operating hours of the schools) although some children also did not finish because they became bored due to the repetitiveness of the staircase-based task. With an average duration of 25 min (occurring after about 15 min of the two other tasks in this study) it was also the longest task of all our paradigms and was very demanding for participants. To statistically test these observations, we calculated Bonferroni-corrected t-tests that compared each age group to adult data in each task. We found significant differences in completeness between adult data and children of the ages of 3, 4, and 5 in Visual Search, 1 in Extinction Learning, and 1 in Visuo Spatial RT. Those data points that were indicated as significantly different from adult data were denoted with empty symbols in Figure 8, those with no difference with filled symbols. For the sake of brevity, detailed t-test results are omitted in this and the following sections, but can be found in supplemental data. Taken together, our data suggests that from the age of 2 onwards, children had sufficient tablet skills and motivation to complete the tasks they were presented with.

### Accuracy

**Figure 9:**
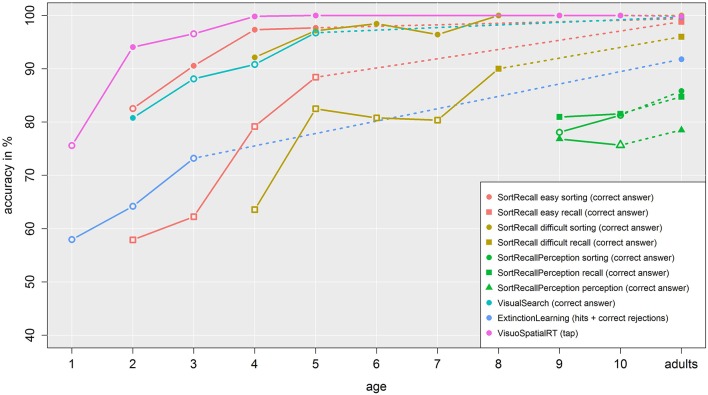
**Accuracy data**. The plot shows the accuracy rate in percent over age. Each line represents a task and the same colors denote sub tasks from the same study, which are differentiated by symbol. Dashed lines connect child data with adult data. Empty symbols denote a significant difference between children and adult data.

While task-specific effects in our studies are most certainly related to age, here we want to investigate the general ability of our participants to understand and correctly handle the task they were given when compared to adult subjects. This metric was defined through correct answers for studies 1–4, while study 5 (Extinction Learning) used the correctness rate based on hits and correct rejections compared to false alarms and misses. Study 6 data was defined as accurate when a tap occurred within the 10 s timeout limit. In Figure 9 we plotted these results per age for each study. Independent of absolute, task-dependent values, the trend of our accuracy data is clearly visible: in each task, younger children exhibit a higher error rate than their older counterparts, which in turn are slightly below adult level. Especially in harder tasks (e.g., recall compared to sorting or the moving stimuli in Extinction Learning compared to the static stimulus in Visuo Spatial RT), the difference is more prominent. Additionally, the accuracy rate depends on the difficulty of stimuli, as can be seen when comparing the tasks in Sort Recall easy, Sort Recall difficult and Sort Recall Perception. Those three studies used the same design and tasks, but included increasingly difficult stimuli. Importantly, none of the accuracy dependent tasks (recall, perception, Visual Search, Extinction Learning) reached ceiling or floor level for our young participants, which allows their use in investigating task-specific effects.

Statistically, Bonferroni-corrected t-tests indicated a significant difference between adults and children aged 2 for Sort Recall easy sorting, 2, 3, 4, and 5 for Sort Recall easy recall, 4, 5, 6, and 7 for Sort Recall difficult recall, 9 and 10 for Sort Recall Perception sorting, 10 for Sort Recall Perception perception, 3, 4, and 5 for Visual Search and 1, 2, and 3 for Extinction Learning, and 1, and 3 for Visuo Spatial RT. As before, we indicated these results as empty symbols; detailed results can be found in a supplemental table. Briefly, accuracy steadily increased over age but, importantly, even the youngest children performed above chance while the older children still performed below ceiling (with the exception of Visuo Spatial RT, which was designed to be as pure a measure of simple response time as possible). These results argue that all tasks were at an appropriate difficulty for their respective age ranges.

### Reaction times

**Figure 10:**
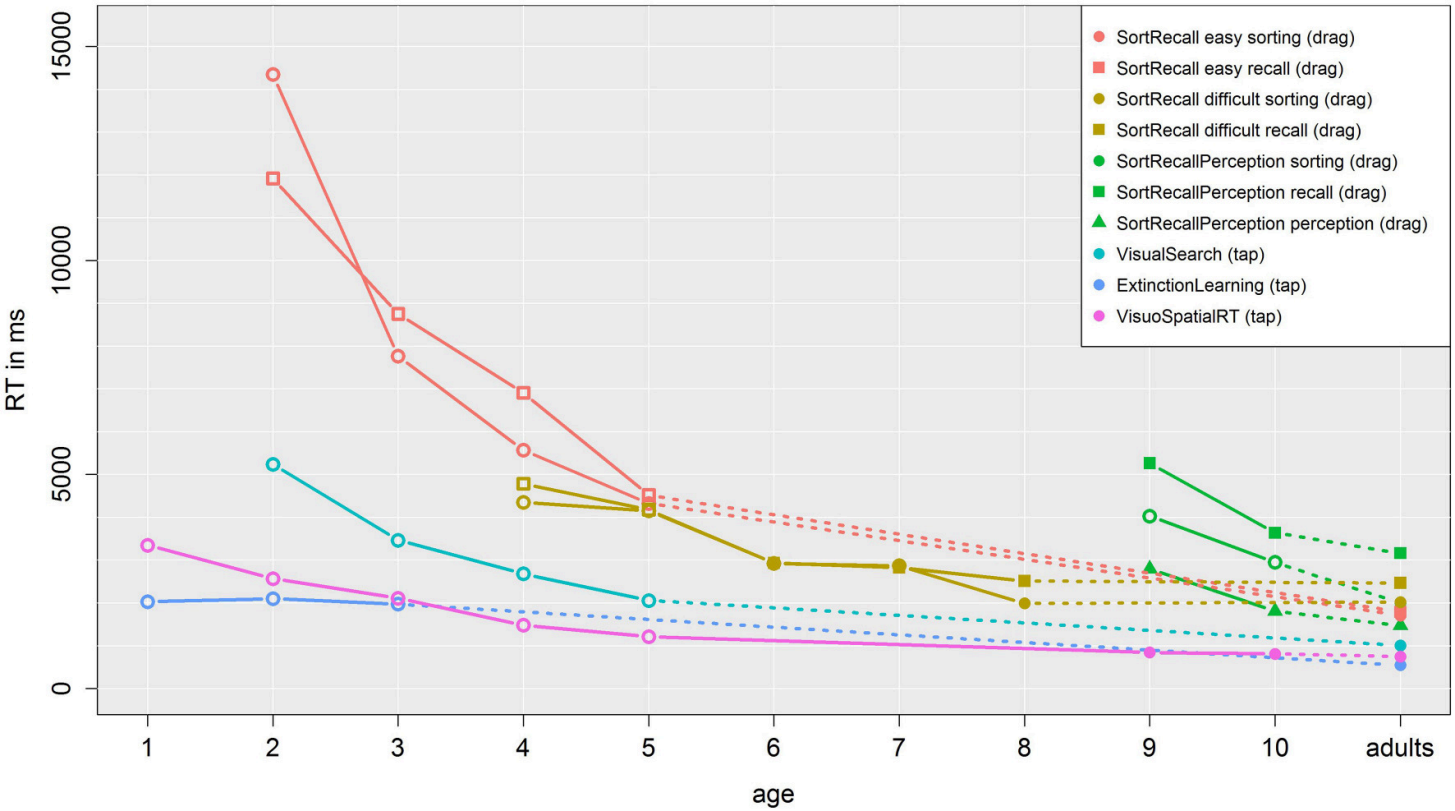
**Reaction time data**. Each line represents one task, with same colors denoting sub-tasks from the same study. Dotted lines connected children and adult data, while empty symbols indicate a significant difference from the age and adult data.

Response time was defined as either the duration between appearance of the stimulus and the drag motion onto a respective target area (studies Sort Recall and Sort Recall Perception) or as the duration between the appearance of the stimulus until a tap on a target or distractor (studies Visual Search, Extinction Learning, and Visuo Spatial RT). Figure 10 shows these results for each task plotted over age. In all tasks, response time generally decreases across development. However this change is not linear; after the age of 5, children's response times quickly converge toward adult values. Notably, there is a very clear and consistent differentiation between the three sub tasks of Sort Recall Perception across different ages, including adults. The cognitively least demanding task (perception) exhibits the fastest reaction time, followed by the sorting task, which requires slight cognitive processing, with the cognitively most demanding task, recall, exhibiting response times that are almost 2 s longer. Importantly, these response times all require the same motor action (dragging and dropping an image). Additionally, all tap tasks show a clear linear decrease in response time over age from 2000 ms (Extinction Learning, 1-year-olds), 3000 ms (Visuo Spatial RT, 1-year-olds), and 5000 ms (Visual Search, 2-year-olds) to about 1000 ms (adults).

Bonferroni-corrected *t*-tests show a significant difference in response time between adults and children at the age of 2, 3, 4, and 5 for Sort Recall easy sorting, 2, 3, 4, and 5 for Sort Recall easy recall, 4, 5, 6, and 7 for Sort Recall difficult sorting, 4, and 5 for Sort Recall difficult recall, 9, and 10 for Sort Recall Perception sorting, 2, 3, 4, and 5 for Visual Search, 1, 2, and 3 for Extinction Learning, and 1, 2, 3, 4, and 5 for Visuo Spatial RT. Details can be found in the supplement material and significant differences are indicated in the graph as empty symbols. In short, the speed of giving a correct answer increases over age. Depending on the task, 8- to 10-year-olds are already almost as fast as adults. For more details, with a specific focus on response times for tap actions, see the following section.

### Visuo-spatial results

**Figure 11:**
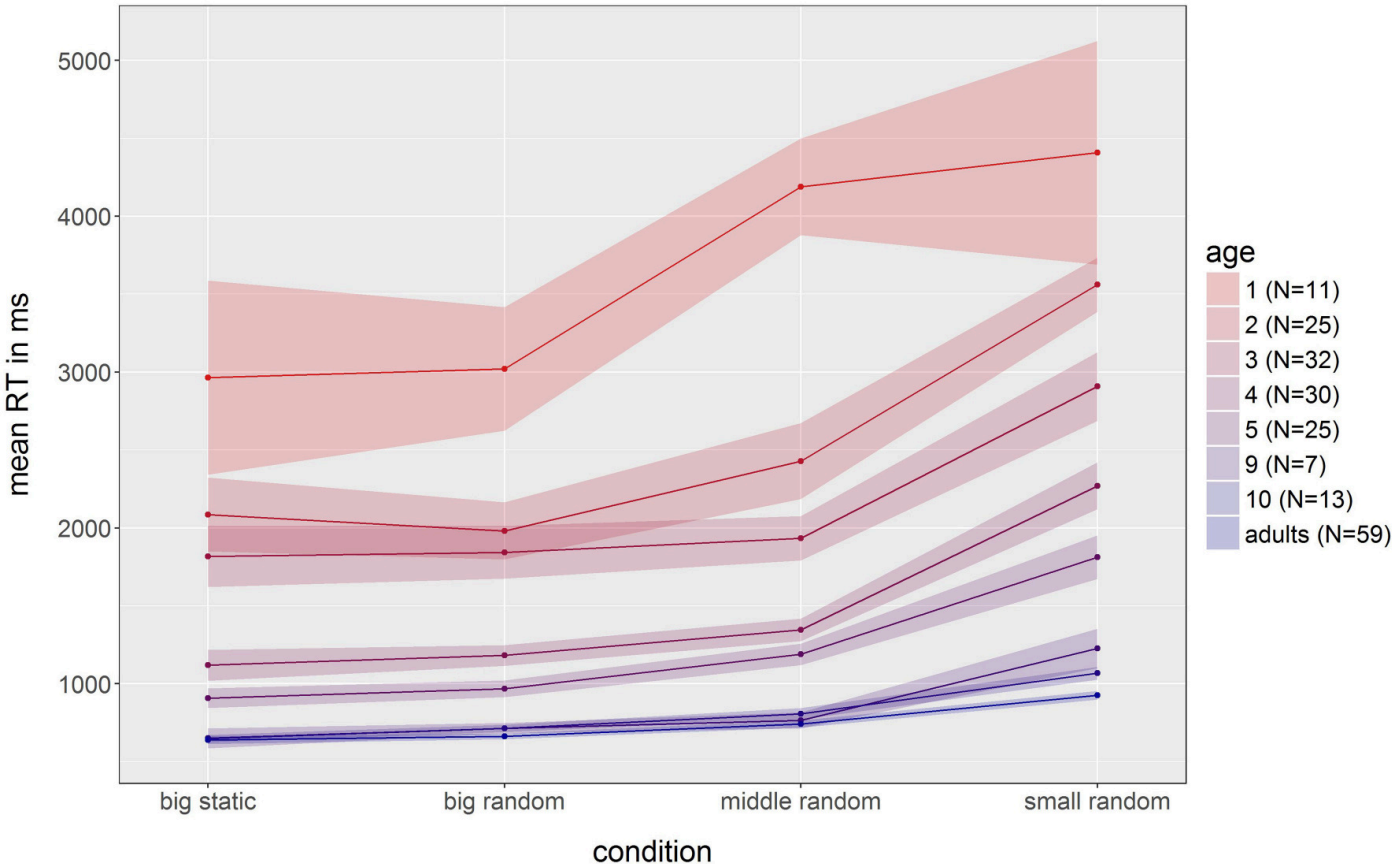
**Detailed results of study 6 (Visuo Spatial RT)**. Each line represents the results from one age group. Standard error is represented by the semi-transparent ribbon of the same color. The first condition started with a big, static, centrally presented stimulus that then decreased in size (big, middle, small) and appeared at a random position in the other three conditions. Colors change from red (youngest participants) to blue (adults).

To further investigate the ability of children to tap on touch screens, we analyzed the results of study 6 (Visuo Spatial RT) in more detail. Figure 11 shows the general increase in reaction time in all ages over condition, where the easiest condition was a big green frog presented centrally, which then appeared in a random position in condition 2, before decreasing twice in size in conditions 3 and 4. Only data sets that had at least one answer were analyzed and data were further processed by removing training trials and misses. We find three noteworthy results: First, there is a very clear increase in speed over age. With each subsequent age group, the reaction times in all conditions become faster, up to a plateau at adult level by around 9 years of age, as reflected in significant Bonferroni corrected *t*-tests between 1- to 5-year-olds and adults (all *p* < 0.01), but no significant differences between 9- and 10-year-olds and adults (all *p* > 0.05). On average, 1-year-old participants exhibited reaction times that were 390% higher than those of adults, followed by a 238% increase for 2-year-olds, 186% for 3-year-olds, 99% for 4-year-olds, and 64% for 5-year-olds. Second, the data from older children, in this case 9- and 10-year-olds (13% and 9% lower speed respectively), needs to be viewed in more detail. In the first three conditions, reaction time matches adult level (between and 1% and 9% slower), but the 4th and therefore hardest condition with the smallest stimulus still shows a significant decrease in speed compared to adults (32% higher reaction times for 9-year-olds and 15% higher reaction times for 10-year-olds). Lastly, the variance in reaction times also decreases over age. When comparing data from 2-year-olds and 4-year-olds, with the same group size, there is a visible decrease in standard error.

Taken together, we argue that there is a clear, easily measurable development in the speed of motor reactions to visual stimuli presented on touch screens across at least the whole age range tested here (1- to 10-year olds) and probably beyond. Despite this, when using reasonably sized stimuli, we were able to obtain equivalent reaction times for 9- and 10-year-olds as for adults. In sum, these results support the general assumption that motor control is still developing across childhood and that reaction speed is highly dependent on age. Here, we also show that a simple RT test on a tablet device can measure these developmental changes so that cognitive researchers can take motor differences into account when assessing development in their main task of interest.

### Summary

**Figure 12:**
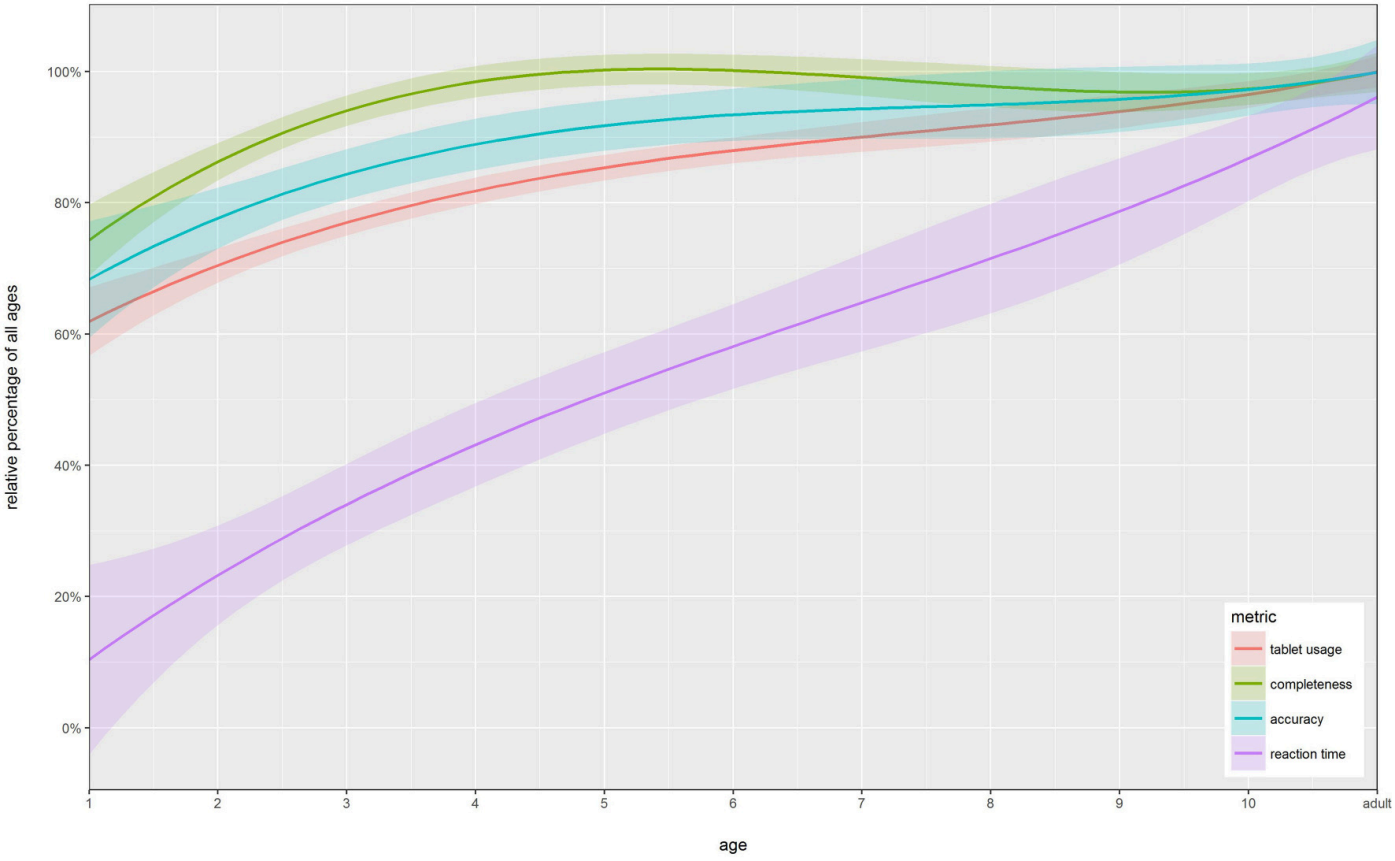
**Summary of all metrics**. Cumulative metrics of all study's plotted over age. Tablet usage has been weighted to be quantifiable. Third degree polynomial smoothing has been applied, and standard error is shown as a semi-transparent area around each line.

To create a one-glance summary of all metrics over all studies, we calculated a cumulative relative measure of tasks. More specifically, for the metrics tablet usage, completeness and accuracy we calculated the maximal value for each sub task and related all other results within this sub task by calculating each as a percentage of the max. To do so for the tablet usage items, we weighted them beforehand with 1 for “no experience,” 2 for “little experience,” and 3 for “much experience.” For the response time data, we first inverted the values before applying the same method. The third degree polynomial smoothed results of this approach can be found in Figure 12.

As can be seen from the graph, tablet usage strongly increases between the ages of 1 and 5 to a plateau that is about 80% of adult data. Similarly, completeness shows a sharp increase between 1 and 4, after which on it stays at close to adult level. The local maximum of this metric around the age of 5 is due to the nature of our studies: 1 (Sort Recall easy), 2 (Sort Recall difficult), and 4 (Visual Search), all conducted with 5-year-olds, had a very high completion rate, while study 3 (Sort Recall Perception), conducted with 9- and 10-year-olds, included exhaustive sub-tasks like the perception task (see 3.2 for details) and had a slightly lower completion rate. Examining accuracy, we find it starts out at a very high level of 70% of adult level and therefore has the smallest increase over age of all our metrics, which argues that our paradigms exhibit similar difficulty across age-groups. This confirms our assumption that our tasks were appropriately difficult for each age range, while still revealing developmental change over age. The last metric, response time, shows the largest increase over age. On average, 1-year-olds exhibit about 15% of the speed at which adults are able to perform the tasks. This difference becomes linearly smaller over age, as mentioned before (see Section Visuo-spatial results for details).

## Discussion

In this work, we evaluated six studies that used touchscreen tablets as data acquisition devices with children between the age of 1 and 10 as well as adults. We used four metrics—tablet usage, completeness, accuracy, and reaction time—to evaluate whether tablets are an appropriate and effective method to conduct experiments and collect data in developmental psychology. In sum (Figure 12), we found that children have enough experience and enough motor control to use a tablet already at the age of 2 (Figure 7), combined with enough persistence and will to complete studies designed to be age appropriate (Figure 8). From the age of 5 onwards, in most tasks, participants are at ceiling for completeness, while differences in accuracy still allow us to measure developmental effects (Figure 9). The fourth metric, response time, can be seen to linearly improve over age until participants reach the age of 9 or 10, at which point they perform, on most tasks, at adult-like speeds (Figures 10, 11). In short, while we find slight—partially task-specific—differences in the metrics we investigated, tablets seem to be a promising tool with which to acquire experimental data and begin to close the aforementioned methodological gap in developmental psychology.

As stated in Section Hardware and Software, we deployed two different types of tablets, a browser, and a combination of HTML and JavaScript to present stimuli and record input from our participants. There were two main motivations for using web technology as software in presenting our experiments. First, due to web technology's native ability to interpret and process touch events, it allowed us to implement the experiments quickly and easily, without the need to program additional interpreters or similar. Second, using web technology allows researchers to publish experiments online, making it possible for parents to participate with their children from home. Such a scenario would make the collection of large and diverse data sets much easier. However, the use of web technology comes with concerns about data security, questions about measurement reliability (Frank et al., 2016) across different platforms and screen sizes, and the need for reliable, consistent internet access during data acquisition. In the present experiment, two of these concerns were solved by using a locally installed webserver. Removing the need for internet access greatly reduced potential concerns regarding how data is transmitted and stored, as we saved the data directly on each device. Getting rid of the need to have a Wi-Fi or data connection also allowed us to conduct research in a variety of locations; a great gain in freedom, especially when compared to static, lab-based experimental computers that are the current common standard in most areas of cognitive psychology. Measurement reliability in regard to timing accuracy is a widely discussed topic in other areas of psychology. However, recent studies show that effects can reliably be reproduced using web technology, especially when using within-subject designs, as was done here (e.g., Crump et al., 2013). Finally, scientists need to be cautious with the robustness of their paradigms when designing for the tablet. Unintended gestures (e.g., dragging instead of tapping, or using two hands and simultaneously interacting with the screen) might lead to technical issues or spurious between-subject (or age) differences. In our case, this was observable in study 5, 1-year-olds surprisingly managed to crash the application in several cases due to their “taps” being rather uncoordinated hitting on the screen with both hands in parallel. Thus, precautionary measures need to be employed to allow only the actions that are intended to be measured and exclude all other possible responses, therefore making experiments “foolproof” (see Section Hardware and Software for details). Despite these cautions, in sum, we found web technology implemented on tablet devices to be a reliable and easy way to employ different kinds of paradigms.

Although general tablet usage was not our main focus, the results of our questionnaire show a clear trend toward increasing tablet usage with age: While some children between the ages 1 and 4 did not have any contact with touchscreen devices and only some used them on a regular basis, the proportion slowly but steadily became inverted from the age of 5 onwards. Our data led us to infer that nowadays, from the age of 10 onwards, almost all children as well as adults have regular, intensive interaction with touchscreen devices. This has obvious implications for conducting experiments: Children below 10 years might still be in the learning process of how to intentionally operate a touch screen, with 1-year-olds definitely having large gaps of knowledge while older children generally know how to coherently interact with the screens. More extensive research on the possible effects of unfamiliarity on acquired data should be done in order to be able to differentiate between paradigms in which expert participants might have an advantage and those where even naïve users are on equal footing. In general, our data suggests that 2-year-olds have enough experience with touch-screen devices to successfully interact with them in an experimental paradigm where simple touch responses are used. Yet, we found our three-point scale not able to differentiate tablet usage in a detailed manner. A continuous scale (e.g., hours per week) would rely less on the interpretation of participants and allow for additional correlation analysis, and therefore we suggest to employ such a scale in future investigations.

Our analyses of “completeness” support the notion that age is a good indicator of effective interaction with tablets. One-year-olds exhibit a 20–30% lower completeness rate than 2-year-olds in the same study, which quickly rises above 95% completeness by the age of 4. That the completeness of 2-year-olds in Visual Search did not yield significant results, despite being of a lower value of 3-year-olds that were significantly different to adults is attributed to the lower sample size. In general, the reasons why the youngest participants are not able or willing to participate for the full duration vary. In study 6 for example, some children were frightened of the green jumping frog we used as a stimulus. This was a surprise to us, as it was intentionally designed to be attractive for toddlers. Other children simply did not understand the task or the necessary actions (i.e., tapping the frog), despite experimenter's demonstrations during the training trials. These participants played with the tablet itself instead of paying attention to the screen and following the instructions. Combining these findings with the results of our questionnaire, we can conclude that 1-year-olds might not produce reliably robust results due to their inexperience with touch screens and inability or unwillingness to engage with the task. For older children, on the other hand, the main limiting factor was boredom. If participants were repeatedly presented with monotonous tasks, like the staircase-based perception task in our study 3, some children became uncomfortable and tired and wanted to quit the study. In addition to lack of motivation, we also experienced extrinsic limitations. Due to the duration of this task, sometimes our experimental times exceeded the time limits set by the teachers at the school or by the end of the school day. Thus, in general, we would suggest limiting experiments, whenever possible, to experimental times below 30 or even 15 min, even in older children. Still, we were pleasantly surprised that even in 1-year-olds we achieved data acquisition rates of around 65%, rising to at least 85% from the age of 2 onwards. This clearly argues that the necessary actions themselves—tapping, dragging—as well as the cognitive requirements for the tasks—sorting, recalling, perception, and visual search—are suitable for these ages. The only potential pitfall regarding completeness of data acquisition seems to be too long and/or repetitive tasks, which should be carefully considered.

Whether the tasks were also executed appropriately was investigated through our third metric, accuracy. Obviously accuracy is the task-relevant metric in nearly all studies, thus we did not expect the young participants to achieve the same level as adult subjects. Still, it would have been concerning if they produced error rates such that they were performing at chance levels. We found an increase in accuracy rates over age in all tasks of all studies. Yet, while completeness data shows two big jumps toward ceiling in very young children, in accuracy data we found a rather sequential increase over all ages. The slowly but steadily higher accuracy of participants over age argues that our paradigms were at an appropriate difficulty level and were well understood by the participants. The lack of floor and ceiling effects further suggests that developmental processes can be uncovered by further investigation of task-specific effects. Yet, when thinking about investigating task-specific effects, one has to consider that our data is partially based on a low number of participants. The primary analysis of error rates that we presented here should not be performed in more detail, especially when considering developmental processes, with a sample size *N* < 10. Nevertheless, this finding complements our completeness data in showing that the cognitive requirements we employed in these tasks—sorting of stimuli into categories, recalling seen stimuli from memory, differentiating between stimuli based on perception, and searching for specific categories among distractors—were all suitable for the children.

With regard to reaction times, we combined two ways of analyzing the data. First, like the other three metrics, we investigated each task over age. Here, we found that on average reaction time decreased with age, as expected. Tapping speed was already close to adult level even in the very young participants, while dragging and dropping took much longer for 2- and 3-year-old children than for adults. Thus, the more complicated the expected action response is, the higher the potential difference in reaction times between ages will be. This clearly suggests that response characteristics need to be considered when designing paradigms. As a supplemental analysis, we assessed the data from study 6 in more detail and found increasing response speeds up to 9- and 10-year-olds, who were only slower than adults in a condition with a very small stimulus. Logistically, we would recommend providing targets that are big enough for children to tap and drag easily to avoid frustration and contaminating cognitive data with motor effects. Nevertheless, these findings point to the importance of identifying a threshold at which adults and children operate on a common ground in senso-motoric ability. When considering taking reaction times as an experimental metric, one has to assure that differences do consist of cognitive differences on the one, but importantly motoric disadvantages on the other hand. Unfortunately, this important differentiation—can the decrease in response times with age across tasks can solely be attributed to motor abilities or a general increase in cognitive and attentional capabilities—cannot fully be covered by our data. To investigate this issue, we would need to supplement our general reaction time task with another task that sequentially increases cognitive load—over all ages. In this case, we could single out whether an increase of reaction time is static, or becomes larger through increased mental resource requirements.

Lastly, we wanted to take another look at the advantages of using tablets in developmental psychology. In all our studies, we found the children (except some percentage of 1-year-olds) to be very engaged and naturally interested in interacting with the touch screens. This confirms results of previous studies (e.g., Frank et al., 2016), but also suggests that tablets may provide a way for closing the methodological gap presented before: Essentially we can, through tablet-mediated experimentation, conduct psychophysical studies from the age of 2 onwards with only a few limitations with regards to length and stimulus material. Children are fascinated by the “gamified” experiments, easily become engaged and decide by themselves—sometimes very clearly—when they have had enough. Combining these characteristics with the portability of touch screen tablets and the ease of data acquisition across many places with many children—e.g., day care, kindergarten, schools or museums—yields high amounts of data with relatively little effort in a pleasurable way for both experimenters and participants. Additionally, this kind of flexible testing in comfortable environments may be especially helpful when thinking about acquiring data from special populations —for instance children with autism or other social impairments for whom role-plays or eye tracking paradigms may be unsuitable. All these advantages do not only apply for classical research studies, but also open up the field of online data acquisition for young children by publishing tablet-based online experiments that can be “played” from home; without the need for lengthy instructions or the presence of an experimenter.

Summing up, through an array of 6 experiments we found that tablet-based experimentation might prove to be an invaluable tool for conducting research with children. Keeping tasks interactive, below 15 min in length, and based on metrics like error rates should produce reliable and robust data from the age of 2 onwards. Mobility, low cost and easy implementation put touch screen paradigms on par with already established methods like role-plays and eye tracking. Further work should investigate potential effects of tablet familiarity on results, the acceptable limits of children's endurance, potential interactions between the development of cognitive load and motor development in reaction time measures, and the publishing and distribution of such experiments through the internet to the wider public. Despite the long road ahead, we already can recommend integrating new technologies in developmental research and the use of tablet-based experimentation to obtain data we might not be able to acquire otherwise.

## Author contributions

KSe: Programming, design, analysis of all studies. MN: Design of all studies. KSo: Design, material, data acquisition of study 1. RR: Design, material, data acquisition of study 2. LM: Design, data acquisition of study 3 and data acquisition of study 6. HP: Design, material, data acquisition of study 4 and data acquisition of study 6. ST: Design, material, data acquisition of study 5 and data acquisition of study 6. TM: Design of study 4. KK: Design, material of studies 1 and 3, piloting of study 3. SW: Design, analysis of all studies. All authors participated in writing and approved the final version of the paper.

## Funding

This work was supported by a PhD scholarship of the German Academic Scholarship Foundation (Studienstiftung des deutschen Volkes) to KS and grants from the German Research Foundation (Deutsche Forschungsgemeinschaft, WE 5802/1-1) and the Mercator Research Center Ruhr (AN-2014-0056) to SW.

### Conflict of interest statement

The authors declare that the research was conducted in the absence of any commercial or financial relationships that could be construed as a potential conflict of interest.
