# Supplementary material for: U Can Touch This: How Tablets Can Be Used to Study Cognitive Development
Source: Front Psychol. 2016 Jul 7;7:1021. doi: 10.3389/fpsyg.2016.01021 (PMC4935681; doi:10.3389/fpsyg.2016.01021)
Supplement: Supplementary file 1 [file DataSheet1.DOCX]

Supplementary Material

­­­­

U Can Touch This:
Using Tablets to Study Cognitive Development in Young Children

Kilian Semmelmann^1*^, Marisa Nordt^1^, Katharina Sommer^1^, Rebecka Röhnke^1^, Luzie Mount^1^, Helen Prüfer^1^, Sophia Terwiel^1^, Tobias W. Meissner^1^, Kami Koldewyn^2^, Sarah Weigelt^1^

^1^Department of Developmental Neuropsychology, Institute of Psychology, Ruhr-University Bochum, Germany

^2^School of Psychology, Bangor University, Bangor, United Kingdom

*** Correspondence:**Kilian Semmelmann, Ruhr-Universität Bochum, Universitätsstr. 150, 44801 Bochum
kilian.semmelmann@rub.de

# Data

## Tablet Usage

|  | 1 | 2 | 3 | 4 | 5 | 6 | 7 | 8 | 9 | 10 | adults |
| --- | --- | --- | --- | --- | --- | --- | --- | --- | --- | --- | --- |
| no experience | 0.29 | 0.06 | 0.10 | 0.05 | 0.00 | 0.00 | 0.00 | 0.00 | 0.00 | 0.00 | 0.00 |
| little experience | 0.65 | 0.69 | 0.50 | 0.50 | 0.41 | 0.15 | 0.57 | 0.30 | 0.14 | 0.00 | 0.02 |
| much experience | 0.06 | 0.25 | 0.40 | 0.45 | 0.59 | 0.85 | 0.43 | 0.70 | 0.86 | 1.00 | 0.98 |

## Completeness

| task | age | completeness in % |
| --- | --- | --- |
| ExtinctionLearning | 1 | 68.24 |
| ExtinctionLearning | 2 | 87.17 |
| ExtinctionLearning | 3 | 83.33 |
| ExtinctionLearning | adults | 100.00 |
| SortRecall difficult recall | 4 | 100.00 |
| SortRecall difficult recall | 5 | 100.00 |
| SortRecall difficult recall | 6 | 100.00 |
| SortRecall difficult recall | 7 | 100.00 |
| SortRecall difficult recall | 8 | 100.00 |
| SortRecall difficult recall | adults | 100.00 |
| SortRecall difficult sorting | 4 | 100.00 |
| SortRecall difficult sorting | 5 | 100.00 |
| SortRecall difficult sorting | 6 | 100.00 |
| SortRecall difficult sorting | 7 | 100.00 |
| SortRecall difficult sorting | 8 | 100.00 |
| SortRecall difficult sorting | adults | 100.00 |
| SortRecall easy recall | 2 | 80.56 |
| SortRecall easy recall | 3 | 100.00 |
| SortRecall easy recall | 4 | 100.00 |
| SortRecall easy recall | 5 | 100.00 |
| SortRecall easy recall | adults | 97.14 |
| SortRecall easy sorting | 2 | 80.28 |
| SortRecall easy sorting | 3 | 100.00 |
| SortRecall easy sorting | 4 | 100.00 |
| SortRecall easy sorting | 5 | 100.00 |
| SortRecall easy sorting | adults | 97.14 |
| SortRecallPerception perception | 9 | 91.43 |
| SortRecallPerception perception | 10 | 92.31 |
| SortRecallPerception perception | adults | 100.00 |
| SortRecallPerception recall | 9 | 100.00 |
| SortRecallPerception recall | 10 | 100.00 |
| SortRecallPerception recall | adults | 99.63 |
| SortRecallPerception sorting | 9 | 100.00 |
| SortRecallPerception sorting | 10 | 100.00 |
| SortRecallPerception sorting | adults | 100.00 |
| VisualSearch | 2 | 77.78 |
| VisualSearch | 3 | 78.99 |
| VisualSearch | 4 | 91.11 |
| VisualSearch | 5 | 90.28 |
| VisualSearch | adults | 100.00 |
| VisuoSpatialRT | 1 | 64.30 |
| VisuoSpatialRT | 2 | 91.68 |
| VisuoSpatialRT | 3 | 95.72 |
| VisuoSpatialRT | 4 | 99.93 |
| VisuoSpatialRT | 5 | 96.15 |
| VisuoSpatialRT | 9 | 100.00 |
| VisuoSpatialRT | 10 | 100.00 |
| VisuoSpatialRT | adults | 99.93 |

## Accuracy

| experiment | age | accuracy in % |
| --- | --- | --- |
| ExtinctionLearning | 1 | 57.94 |
| ExtinctionLearning | 2 | 64.19 |
| ExtinctionLearning | 3 | 73.20 |
| ExtinctionLearning | adults | 91.78 |
| SortRecall difficult recall | 4 | 63.57 |
| SortRecall difficult recall | 5 | 82.50 |
| SortRecall difficult recall | 6 | 80.77 |
| SortRecall difficult recall | 7 | 80.36 |
| SortRecall difficult recall | 8 | 90.00 |
| SortRecall difficult recall | adults | 96.00 |
| SortRecall difficult sorting | 4 | 92.14 |
| SortRecall difficult sorting | 5 | 97.14 |
| SortRecall difficult sorting | 6 | 98.46 |
| SortRecall difficult sorting | 7 | 96.43 |
| SortRecall difficult sorting | 8 | 100.00 |
| SortRecall difficult sorting | adults | 100.00 |
| SortRecall easy recall | 2 | 57.89 |
| SortRecall easy recall | 3 | 62.22 |
| SortRecall easy recall | 4 | 79.17 |
| SortRecall easy recall | 5 | 88.43 |
| SortRecall easy recall | adults | 98.81 |
| SortRecall easy sorting | 2 | 82.54 |
| SortRecall easy sorting | 3 | 90.56 |
| SortRecall easy sorting | 4 | 97.35 |
| SortRecall easy sorting | 5 | 97.69 |
| SortRecall easy sorting | adults | 99.40 |
| SortRecallPerception perception | 9 | 76.83 |
| SortRecallPerception perception | 10 | 75.67 |
| SortRecallPerception perception | adults | 78.49 |
| SortRecallPerception recall | 9 | 80.95 |
| SortRecallPerception recall | 10 | 81.54 |
| SortRecallPerception recall | adults | 84.75 |
| SortRecallPerception sorting | 9 | 78.10 |
| SortRecallPerception sorting | 10 | 81.28 |
| SortRecallPerception sorting | adults | 85.83 |
| VisualSearch | 2 | 80.77 |
| VisualSearch | 3 | 88.13 |
| VisualSearch | 4 | 90.82 |
| VisualSearch | 5 | 96.75 |
| VisualSearch | adults | 99.71 |
| VisuoSpatialRT | 1 | 75.58 |
| VisuoSpatialRT | 2 | 94.07 |
| VisuoSpatialRT | 3 | 96.56 |
| VisuoSpatialRT | 4 | 99.83 |
| VisuoSpatialRT | 5 | 100.00 |
| VisuoSpatialRT | 9 | 100.00 |
| VisuoSpatialRT | 10 | 100.00 |
| VisuoSpatialRT | adults | 99.83 |

## Reaction Time

| experiment | age | rt in ms |
| --- | --- | --- |
| ExtinctionLearning | 1 | 2026 |
| ExtinctionLearning | 2 | 2101 |
| ExtinctionLearning | 3 | 1970 |
| ExtinctionLearning | adults | 543 |
| SortRecall difficult recall | 4 | 4784 |
| SortRecall difficult recall | 5 | 4177 |
| SortRecall difficult recall | 6 | 2934 |
| SortRecall difficult recall | 7 | 2822 |
| SortRecall difficult recall | 8 | 2512 |
| SortRecall difficult recall | adults | 2468 |
| SortRecall difficult sorting | 4 | 4349 |
| SortRecall difficult sorting | 5 | 4148 |
| SortRecall difficult sorting | 6 | 2923 |
| SortRecall difficult sorting | 7 | 2865 |
| SortRecall difficult sorting | 8 | 1989 |
| SortRecall difficult sorting | adults | 2015 |
| SortRecall easy recall | 2 | 11913 |
| SortRecall easy recall | 3 | 8749 |
| SortRecall easy recall | 4 | 6908 |
| SortRecall easy recall | 5 | 4515 |
| SortRecall easy recall | adults | 1787 |
| SortRecall easy sorting | 2 | 14341 |
| SortRecall easy sorting | 3 | 7761 |
| SortRecall easy sorting | 4 | 5564 |
| SortRecall easy sorting | 5 | 4323 |
| SortRecall easy sorting | adults | 1707 |
| SortRecallPerception perception | 9 | 2795 |
| SortRecallPerception perception | 10 | 1805 |
| SortRecallPerception perception | adults | 1472 |
| SortRecallPerception recall | 9 | 5263 |
| SortRecallPerception recall | 10 | 3635 |
| SortRecallPerception recall | adults | 3163 |
| SortRecallPerception sorting | 9 | 4027 |
| SortRecallPerception sorting | 10 | 2948 |
| SortRecallPerception sorting | adults | 2001 |
| VisualSearch | 2 | 5238 |
| VisualSearch | 3 | 3465 |
| VisualSearch | 4 | 2679 |
| VisualSearch | 5 | 2057 |
| VisualSearch | adults | 1007 |
| VisuoSpatialRT | 1 | 3347 |
| VisuoSpatialRT | 2 | 2567 |
| VisuoSpatialRT | 3 | 2108 |
| VisuoSpatialRT | 4 | 1480 |
| VisuoSpatialRT | 5 | 1211 |
| VisuoSpatialRT | 9 | 840 |
| VisuoSpatialRT | 10 | 810 |
| VisuoSpatialRT | adults | 743 |

## Visuo-Spatial RT data

| condition | age | rt in ms | SEM |
| --- | --- | --- | --- |
| big random | 1 | 3021 | 396 |
| big static | 1 | 2965 | 623 |
| middle random | 1 | 4190 | 310 |
| small random | 1 | 4407 | 717 |
| big random | 2 | 1981 | 183 |
| big static | 2 | 2086 | 236 |
| middle random | 2 | 2429 | 244 |
| small random | 2 | 3561 | 174 |
| big random | 3 | 1844 | 169 |
| big static | 3 | 1818 | 197 |
| middle random | 3 | 1934 | 142 |
| small random | 3 | 2908 | 221 |
| big random | 4 | 1182 | 67 |
| big static | 4 | 1119 | 98 |
| middle random | 4 | 1347 | 72 |
| small random | 4 | 2270 | 151 |
| big random | 5 | 969 | 54 |
| big static | 5 | 907 | 63 |
| middle random | 5 | 1189 | 69 |
| small random | 5 | 1812 | 141 |
| big random | 9 | 715 | 33 |
| big static | 9 | 651 | 64 |
| middle random | 9 | 766 | 51 |
| small random | 9 | 1227 | 125 |
| big random | 10 | 714 | 23 |
| big static | 10 | 650 | 23 |
| middle random | 10 | 809 | 37 |
| small random | 10 | 1068 | 41 |
| big random | adults | 663 | 19 |
| big static | adults | 640 | 26 |
| middle random | adults | 742 | 24 |
| small random | adults | 927 | 28 |

# Supplementary t-test results

Enclosed all task-wise Bonferroni corrected t-tests can be found. “NA” values indicated identical data sets.

## Completeness

| task | metric | age | df | t | corr. p | sig |
| --- | --- | --- | --- | --- | --- | --- |
| ExtinctionLearning | completeness | 1 | 16.00 | 3.70 | 0.0059 | * |
| ExtinctionLearning | completeness | 2 | 22.00 | 2.45 | 0.0678 |  |
| ExtinctionLearning | completeness | 3 | 5.00 | 2.19 | 0.2395 |  |
| SortRecall difficult recall | completeness | 4 | NA | NA | NA |  |
| SortRecall difficult recall | completeness | 5 | 13.00 | 1.00 | 1.0000 |  |
| SortRecall difficult recall | completeness | 6 | NA | NA | NA |  |
| SortRecall difficult recall | completeness | 7 | NA | NA | NA |  |
| SortRecall difficult recall | completeness | 8 | NA | NA | NA |  |
| SortRecall difficult sorting | completeness | 4 | NA | NA | NA |  |
| SortRecall difficult sorting | completeness | 5 | 13.00 | 1.00 | 1.0000 |  |
| SortRecall difficult sorting | completeness | 6 | NA | NA | NA |  |
| SortRecall difficult sorting | completeness | 7 | NA | NA | NA |  |
| SortRecall difficult sorting | completeness | 8 | NA | NA | NA |  |
| SortRecall easy recall | completeness | 2 | 29.42 | 2.12 | 0.1687 |  |
| SortRecall easy recall | completeness | 3 | 13.00 | -1.00 | 1.0000 |  |
| SortRecall easy recall | completeness | 4 | 13.00 | -1.00 | 1.0000 |  |
| SortRecall easy recall | completeness | 5 | 13.00 | -1.00 | 1.0000 |  |
| SortRecall easy sorting | completeness | 2 | 29.46 | 2.17 | 0.1539 |  |
| SortRecall easy sorting | completeness | 3 | 13.00 | -1.00 | 1.0000 |  |
| SortRecall easy sorting | completeness | 4 | 13.00 | -1.00 | 1.0000 |  |
| SortRecall easy sorting | completeness | 5 | 13.00 | -1.00 | 1.0000 |  |
| SortRecallPerception perception | completeness | 9 | 6.00 | 1.92 | 0.2061 |  |
| SortRecallPerception perception | completeness | 10 | 12.00 | 2.31 | 0.0790 |  |
| SortRecallPerception recall | completeness | 9 | 15.00 | -1.00 | 0.6663 |  |
| SortRecallPerception recall | completeness | 10 | 15.00 | -1.00 | 0.6663 |  |
| SortRecallPerception sorting | completeness | 9 | NA | NA | NA |  |
| SortRecallPerception sorting | completeness | 10 | NA | NA | NA |  |
| VisualSearch | completeness | 2 | 8.00 | 2.41 | 0.1695 |  |
| VisualSearch | completeness | 3 | 22.00 | 3.48 | 0.0085 | * |
| VisualSearch | completeness | 4 | 29.00 | 3.40 | 0.0080 | * |
| VisualSearch | completeness | 5 | 23.00 | 3.25 | 0.0143 | * |
| VisuoSpatialRT | completeness | 1 | 13.00 | 3.28 | 0.0414 | * |
| VisuoSpatialRT | completeness | 2 | 26.01 | 1.62 | 0.8259 |  |
| VisuoSpatialRT | completeness | 3 | 31.02 | 1.50 | 0.9991 |  |
| VisuoSpatialRT | completeness | 4 | 58.42 | -0.01 | 1.0000 |  |
| VisuoSpatialRT | completeness | 5 | 25.01 | 0.98 | 1.0000 |  |
| VisuoSpatialRT | completeness | 9 | 58.00 | -1.43 | 1.0000 |  |
| VisuoSpatialRT | completeness | 10 | 58.00 | -1.43 | 1.0000 |  |

## Accuracy

| task | metric | age | df | t | corr. p | sig |
| --- | --- | --- | --- | --- | --- | --- |
| ExtinctionLearning | accuracy | 1 | 21.31 | -13.20 | 0.0000 | * |
| ExtinctionLearning | accuracy | 2 | 28.78 | -10.62 | 0.0000 | * |
| ExtinctionLearning | accuracy | 3 | 5.45 | -3.90 | 0.0289 | * |
| SortRecall difficult recall | accuracy | 4 | 15.83 | -6.34 | 0.0001 | * |
| SortRecall difficult recall | accuracy | 5 | 21.72 | -4.92 | 0.0003 | * |
| SortRecall difficult recall | accuracy | 6 | 19.17 | -4.90 | 0.0005 | * |
| SortRecall difficult recall | accuracy | 7 | 18.15 | -4.09 | 0.0034 | * |
| SortRecall difficult recall | accuracy | 8 | 14.85 | -1.91 | 0.3796 |  |
| SortRecall difficult sorting | accuracy | 4 | 13.00 | -1.94 | 0.3720 |  |
| SortRecall difficult sorting | accuracy | 5 | 13.00 | -2.83 | 0.0712 |  |
| SortRecall difficult sorting | accuracy | 6 | 12.00 | -1.76 | 0.5195 |  |
| SortRecall difficult sorting | accuracy | 7 | 13.00 | -1.93 | 0.3767 |  |
| SortRecall difficult sorting | accuracy | 8 | NA | NA | NA |  |
| SortRecall easy recall | accuracy | 2 | 20.08 | -11.91 | 0.0000 | * |
| SortRecall easy recall | accuracy | 3 | 14.50 | -6.02 | 0.0001 | * |
| SortRecall easy recall | accuracy | 4 | 24.18 | -6.55 | 0.0000 | * |
| SortRecall easy recall | accuracy | 5 | 19.65 | -3.48 | 0.0096 | * |
| SortRecall easy sorting | accuracy | 2 | 18.59 | -3.59 | 0.0080 | * |
| SortRecall easy sorting | accuracy | 3 | 14.84 | -2.54 | 0.0918 |  |
| SortRecall easy sorting | accuracy | 4 | 31.92 | -1.76 | 0.3552 |  |
| SortRecall easy sorting | accuracy | 5 | 25.22 | -1.35 | 0.7588 |  |
| SortRecallPerception perception | accuracy | 9 | 13.38 | -2.50 | 0.0525 |  |
| SortRecallPerception perception | accuracy | 10 | 17.92 | -3.12 | 0.0119 | * |
| SortRecallPerception recall | accuracy | 9 | 17.12 | -1.25 | 0.4559 |  |
| SortRecallPerception recall | accuracy | 10 | 23.35 | -0.90 | 0.7581 |  |
| SortRecallPerception sorting | accuracy | 9 | 10.08 | -3.30 | 0.0159 | * |
| SortRecallPerception sorting | accuracy | 10 | 25.83 | -2.63 | 0.0282 | * |
| VisualSearch | accuracy | 2 | 6.01 | -2.87 | 0.1140 |  |
| VisualSearch | accuracy | 3 | 20.09 | -3.16 | 0.0198 | * |
| VisualSearch | accuracy | 4 | 29.40 | -4.32 | 0.0007 | * |
| VisualSearch | accuracy | 5 | 24.21 | -2.78 | 0.0411 | * |
| VisuoSpatialRT | accuracy | 1 | 13.01 | -3.35 | 0.0366 | * |
| VisuoSpatialRT | accuracy | 2 | 26.13 | -2.41 | 0.1637 |  |
| VisuoSpatialRT | accuracy | 3 | 31.94 | -3.36 | 0.0140 | * |
| VisuoSpatialRT | accuracy | 4 | 58.42 | 0.01 | 1.0000 |  |
| VisuoSpatialRT | accuracy | 5 | 58.00 | 1.43 | 1.0000 |  |
| VisuoSpatialRT | accuracy | 9 | 58.00 | 1.43 | 1.0000 |  |
| VisuoSpatialRT | accuracy | 10 | 58.00 | 1.43 | 1.0000 |  |

## Reaction time

| task | metric | age | df | t | corr. p | sig |
| --- | --- | --- | --- | --- | --- | --- |
| ExtinctionLearning | rt | 1 | 17.03 | -10.43 | 0.0000 | * |
| ExtinctionLearning | rt | 2 | 26.25 | -13.68 | 0.0000 | * |
| ExtinctionLearning | rt | 3 | 5.22 | -5.49 | 0.0072 | * |
| SortRecall difficult recall | rt | 4 | 18.55 | -3.82 | 0.0060 | * |
| SortRecall difficult recall | rt | 5 | 18.53 | -2.92 | 0.0448 | * |
| SortRecall difficult recall | rt | 6 | 17.90 | -1.12 | 1.0000 |  |
| SortRecall difficult recall | rt | 7 | 22.00 | -1.24 | 1.0000 |  |
| SortRecall difficult recall | rt | 8 | 12.49 | -0.80 | 1.0000 |  |
| SortRecall difficult sorting | rt | 4 | 17.15 | -4.46 | 0.0017 | * |
| SortRecall difficult sorting | rt | 5 | 17.63 | -4.17 | 0.0030 | * |
| SortRecall difficult sorting | rt | 6 | 20.85 | -2.94 | 0.0393 | * |
| SortRecall difficult sorting | rt | 7 | 21.19 | -3.00 | 0.0337 | * |
| SortRecall difficult sorting | rt | 8 | 12.18 | 0.11 | 1.0000 |  |
| SortRecall easy recall | rt | 2 | 19.39 | -9.64 | 0.0000 | * |
| SortRecall easy recall | rt | 3 | 14.59 | -5.09 | 0.0006 | * |
| SortRecall easy recall | rt | 4 | 22.82 | -5.40 | 0.0001 | * |
| SortRecall easy recall | rt | 5 | 20.33 | -4.36 | 0.0012 | * |
| SortRecall easy sorting | rt | 2 | 18.09 | -3.66 | 0.0072 | * |
| SortRecall easy sorting | rt | 3 | 14.40 | -4.65 | 0.0014 | * |
| SortRecall easy sorting | rt | 4 | 24.23 | -6.59 | 0.0000 | * |
| SortRecall easy sorting | rt | 5 | 20.41 | -5.02 | 0.0002 | * |
| SortRecallPerception perception | rt | 9 | 6.32 | -1.63 | 0.3022 |  |
| SortRecallPerception perception | rt | 10 | 24.71 | -1.73 | 0.1930 |  |
| SortRecallPerception recall | rt | 9 | 10.32 | -2.08 | 0.1257 |  |
| SortRecallPerception recall | rt | 10 | 25.69 | -1.06 | 0.5942 |  |
| SortRecallPerception sorting | rt | 9 | 9.59 | -6.27 | 0.0002 | * |
| SortRecallPerception sorting | rt | 10 | 15.64 | -2.55 | 0.0434 | * |
| VisualSearch | rt | 2 | 6.01 | -4.74 | 0.0127 | * |
| VisualSearch | rt | 3 | 20.41 | -8.78 | 0.0000 | * |
| VisualSearch | rt | 4 | 31.35 | -11.84 | 0.0000 | * |
| VisualSearch | rt | 5 | 25.77 | -9.61 | 0.0000 | * |
| VisuoSpatialRT | rt | 1 | 13.05 | -9.22 | 0.0000 | * |
| VisuoSpatialRT | rt | 2 | 26.35 | -8.31 | 0.0000 | * |
| VisuoSpatialRT | rt | 3 | 31.98 | -9.25 | 0.0000 | * |
| VisuoSpatialRT | rt | 4 | 33.28 | -8.86 | 0.0000 | * |
| VisuoSpatialRT | rt | 5 | 31.53 | -7.02 | 0.0000 | * |
| VisuoSpatialRT | rt | 9 | 7.61 | -1.26 | 1.0000 |  |
| VisuoSpatialRT | rt | 10 | 54.30 | -1.89 | 0.4525 |  |
